# Is There a Role for Colchicine in Acute Coronary Syndromes? A Literature Review

**DOI:** 10.7759/cureus.8166

**Published:** 2020-05-17

**Authors:** Jahanzeb Malik, Nismat Javed, Uzma Ishaq, Umar Khan, Talha Laique

**Affiliations:** 1 Cardiology, Rawalpindi Institute of Cardiology, Rawalpindi, PAK; 2 Internal Medicine, Shifa College of Medicine - Shifa Tameer-E-Millat University, Islamabad, PAK; 3 Hematology and Medical Oncology, Fauji Foundation Hospital, Rawalpindi, PAK; 4 Pulmonary Medicine, University Hospital Limerick, Limerick, IRL; 5 Pharmacology, Lahore Medical and Dental College, Lahore, PAK

**Keywords:** acute coronary syndrome, colchicine, atherosclerosis, ischemic heart disease, inflammation

## Abstract

Inflammation is identified as a keystone of atherosclerosis. This review of the literature explores the unique anti-inflammatory effects of colchicine and summarizes the mechanisms of inflammation in acute coronary syndrome. It outlines other therapeutic strategies employed until now to target coronary inflammation and analyzes the role of colchicine in improving the outcomes of acute coronary syndrome. Despite the existence of guideline-directed medical therapy, there still remains a higher risk for recurrence due to continuous inflammation at remaining vascular sites. Several anti-inflammatory strategies have been employed, but they have not been shown to be beneficial. However, colchicine is becoming increasingly popular in tackling this problem. For this review, databases were searched for trials on the role of colchicine as an anti-inflammatory therapy in acute coronary syndrome.

## Introduction and background

Atherosclerosis is the leading cause of morbidity and mortality worldwide, mostly due to coronary artery disease causing acute coronary syndrome. Guideline-directed medical therapy puts emphasis on the modulation of cardiovascular risk factors like control of diabetes, lipid-lowering therapies, and antiplatelet medications to prevent plaque rupture and thrombus formation. However, increasing evidence is now available that implicates inflammation as the core process for the pathogenesis of atherosclerotic plaque, its changing dynamics, and rupture [1,2]. Patients with a known history of acute coronary syndrome are at an elevated risk for recurrent major adverse cardiovascular events [3]. This is mainly due to the residual inflammatory process in coronary arteries [4].

Statins, used in guideline-directed medical therapy to lower target low-density lipoprotein cholesterol (LDL-C), have been known to possess an anti-inflammatory function in addition to their lipid-lowering effects [5]. Treatment, however, is now shifting towards specific anti-inflammatory approaches to further improve outcomes in these patients. Recent trials have laid emphasis on the hypothesis that highlights the role of inflammation in atherothrombosis. The Canakinumab Anti-inflammatory Thrombosis Outcome Study (CANTOS) found that canakinumab at a dose of 150 mg every three months reduced adverse cardiovascular events in patients with previous myocardial infarction and C-reactive protein (CRP) levels of more than 2 mg/L [6]. Canakinumab is a monoclonal antibody that targets interleukin-1β (IL-1β) [7]. This is an important inflammatory cascade in the interleukin-6 (IL-6) signaling pathway. However, this is not cost-effective and it causes high rates of fatal infections, thus limiting its widespread use.

Colchicine, on the other hand, is a widely available, inexpensive, and tolerable anti-inflammatory medication. It prevents mitosis by inhibiting microtubule polymerization [3,8]. It is mainly used in the management of gout, pericarditis, and familial Mediterranean fever. This review outlines the mechanism of inflammation in acute coronary syndrome and gives an overview of some targeted anti-inflammatory approaches, leading to the evidence that endorses colchicine as an agent for secondary prevention after acute coronary syndrome.

## Review

Methods

To summarize the body of available evidence, a scoping review design was used to incorporate a range of studies and reports in a narrative format. For this purpose, we identified the relevant literature by performing a search of the bibliographic databases of MEDLINE and ClinicalTrials using the keywords “colchicine”, “atherosclerosis”, and “colchicine” and “acute coronary syndrome”. We reviewed the literature for the previous 20 years.

The NOD-like receptor protein 3 (NLRP3) inflammasome and acute coronary syndrome

There is a lot of evidence that local and systemic inflammation plays a pathologic role in acute coronary syndrome. Understanding these mechanisms can lead to newer therapeutic agents and strategies [3,9]. By certain triggers like LDL-C, endothelial activation occurs, releasing pro-inflammatory cytokines, metalloproteinases, and oxygen-free radicals, which increase inflammation and possibly reduce plaque stability by weakening the fibrous cap. Further recruitment of pro-inflammatory cytokines causes inflammatory cell infiltration, which potentiates the pro-coagulant properties of these cells. Rupture of the fibrous cap causes contact of these inflammasomes with blood, leading to thrombosis [9-12].

A specific kind of neutrophils is recognized as an important atheroinflammation contributor causing plaque rupture [13]. Another key player in the innate immune system is the NLRP3 inflammasome. It is a multi-protein compound present in myeloid cells, including neutrophils and eosinophils. Exposure to stress signals is sensed by an NLRP3 receptor, which leads to the assembly of NLRP3 as an adaptor protein apoptosis-associated speck-like substance that contains the caspase recruitment domain [3,14]. Adenosine triphosphate (ATP) activates caspase-1, leading to the secretion of active IL-1β and IL-18. Exposure of neutrophils to stimuli like cholesterol crystals causes activation of NLRP3 inflammasome. This, in turn, promotes the caspase-1-dependent release of two key inflammatory cytokines (IL-1β and IL-18), both of which are predictive of future cardiovascular events and are key mediators in plaque development and destabilization.

IL-1β is a proinflammatory cytokine that causes adhesion of monocytes to vascular surfaces. It induces procoagulant activity via the growth of vascular smooth muscle cells [15]. The activation of IL-1β signals IL-6, which drives the expression of atherothrombosis mediators. It has been found that patients with acute coronary syndrome have the highest transcoronary numbers of IL-1β, IL-6, and IL-18 compared to those with chronic coronary syndromes [15].

This shows that a transformation of atherosclerotic plaque from stable to rupture is mediated by inflammatory cells that destabilize the lesion causing thrombosis and myocardial infarction. However, some studies show that there is widespread activation of neutrophils across the coronary bed in unstable angina, regardless of the culprit lesion [16].

Various agents used for targeting inflammation in acute coronary syndromes

The role of inflammation in acute coronary syndrome has led to several novel therapeutic anti-inflammatory agents being brought to decrease recurrent events under trials. However, despite many promising phase II trials, many of these have not shown any clinical superiority than guideline-directed medical therapy [17].

Statins have pleiotropic anti-inflammatory properties acting synergistically with lipid-lowering. Their mechanism of action includes T-cell activation, leukocyte adhesion, and enhancing endothelial nitric oxide production [5]. Several large trials have shown that statins decrease CRP along with their lipid-lowering effects. The Pravastatin or Atorvastatin Evaluation and Infection Therapy (PROVE-IT) trial enrolled 3,745 patients with acute coronary syndrome to undergo moderate or high-dose statin therapy and concluded that patients with lower CRP had lower cardiovascular events [9]. Similarly, the Improved Reduction of Outcomes; Vytorin Efficacy International Trial (IMPROVE-IT) randomized 18,144 patients after stabilized acute coronary syndrome to simvastatin and measured CRP and LDL-C. Similar to the PROVE-IT trial, there was an almost identical proportion of patients who met the primary composite endpoint (cardiovascular death, acute coronary syndrome, coronary revascularization within 30 days, or nonfatal stroke) in the group that achieved CRP levels of <2 mg/L but LDL-C levels of >1.8 mmol/L [9,18].

Corticosteroids were also used as agents for anti-inflammatory response in acute coronary syndrome. A meta-analysis of 11 trials showed a 26% decrease in mortality; however, the data was insufficient, and there arose a risk of cardiac rupture secondary to impaired wall healing [19]. Apart from aspirin, nonsteroidal anti-inflammatory drugs were found to elevate the risk of cardiovascular events [20].

Certain monoclonal antibodies like inclacumab and pexelizumab were tested but no difference in mortality and morbidity was found [21]. However, one monoclonal antibody that targeted IL-1β, canakinumab, has gained attention recently. Canakinumab Anti-inflammatory Thrombosis Outcome Study (CANTOS) was a randomized trial that evaluated canakinumab along with guideline-directed medical therapy to prevent recurrent cardiovascular events [6]. 10,061 patients with myocardial infarction were included in the study. At 48 months, there was a reduction of CRP at 26% from 50 mg dose and 37% from 150 mg dose. At a follow-up at almost four years, the cardiovascular event was found to be reduced by 15% in the 150-mg group compared to the placebo (P=0.021). There was no difference in all-cause mortality.

Role of colchicine

Colchicine is a widely available, inexpensive anti-inflammatory drug with well-known side effects. Common adverse effects are nausea, vomiting, and diarrhea. Rare side effects include hepatic injury, rhabdomyolysis, hypersensitivity, and blood disorders. Major interactions are with statins and cytochrome P450 3A4 inhibitors [3,22-27]. It acts by microtubule depolymerization and tubulin binding, inhibiting the NLRP3 inflammasome protein complex [28,29]. By monocyte caspase-1 inhibition, it brings about inhibition of colocalization of inflammasome cytoplasmic proteins and inhibition of P2 * 7-mediated pore function, a key step in the NLRP3 response to adenosine triphosphate [30-32]. In patients with acute coronary syndrome, the monocytes exhibited a reduction in secreted levels of IL-1β after they received a low dose of colchicine. Reduction in procaspase-1 was also seen along with trans-coronary caspase-1-mediated cytokines (IL-1β and IL-18) by 88% [33]. These findings confirm that local cholesterol crystal activation causes inflammation within the atherosclerotic plaque, leading to plaque instability and rupture [7,34].

The Low-Dose Colchicine (LoDoCo) trial, which consisted of 532 patients with chronic coronary syndrome who were followed up for three years, showed that colchicine 0.5 mg per day administered along with guideline-directed medical therapy was beneficial in the prevention of adverse cardiovascular events (P=0.001) [35]. In an observational study involving 80 patients with acute coronary syndrome, patients received either guideline-directed medical therapy alone or guideline-directed medical therapy along with colchicine 0.5 mg per day for a year. CT coronary angiography performed at 12 months showed plaque stabilization and change in total atheroma volume. There was a change in low attenuation plaque (LAP) volume with a mean reduction of 41% in the treatment group and 17% in the control group (P=0.008). Similarly, there was a reduction of CRP levels in both groups (37% and 15% in the treatment and control group, respectively) (P=0.001). This study demonstrated that colchicine combined with guideline-directed medical therapy has a more plaque stabilizing effect than guideline-directed medical therapy alone [36].

In an observational study involving 80 patients who had undergone acute coronary syndrome and ischemic stroke, no difference in CRP was found in treatment versus the control group after administering colchicine and following up for 30 days [37]. On the contrary, a study of 64 patients found that low-dose colchicine reduced CRP levels in patients with chronic coronary syndrome [35].

The landmark Colchicine Cardiovascular Outcomes Trial (COLCOT) was a metacentric trial that randomized 4,745 patients with either low-dose colchicine (0.5 mg per day) or placebo. Among patients with recent myocardial infarction on guideline-directed medical therapy, 0.5 mg of colchicine daily reduced the risk of cardiovascular death, acute myocardial infarction, stroke, resuscitated cardiac arrest, or urgent hospitalization for angina requiring revascularization by 23%. Overall rates of adverse events were low with colchicine (0.9% vs 0.4%; P=0.03). This clearly showed the benefit of adding colchicine to guideline-directed medical therapy in patients with acute coronary syndrome [38].

## Conclusions

With the knowledge that inflammation plays a pathologic role in atherosclerosis, new strategies for the treatment and prevention of coronary artery disease can be established. Many anti-inflammatory therapies have been tested during the past decade; however, they have failed to show major differences in outcomes when tested in randomized trials. The role of therapies, particularly colchicine, will hopefully be defined well in future studies.
